# Decreased MicroRNA Is Involved in the Vascular Remodeling Abnormalities in Chronic Kidney Disease (CKD)

**DOI:** 10.1371/journal.pone.0064558

**Published:** 2013-05-22

**Authors:** Neal X. Chen, Kraiwiporn Kiattisunthorn, Kalisha D. O'Neill, Xianming Chen, Ranjani N. Moorthi, Vincent H. Gattone, Matthew R. Allen, Sharon M. Moe

**Affiliations:** 1 Medicine, Indiana University School of Medicine, Indianapolis, Indiana, United States of America; 2 Medicine, Faculty of Siriraj Medical School, Mahidol University, Bangkok, Thailand; 3 Department of Anatomy and Cell Biology, Indiana University School of Medicine, Indianapolis, Indiana, United States of America; 4 Roudebush Veterans Affairs Medical Center, Indianapolis, Indiana, United States of America; University of Louisville, United States of America

## Abstract

Patients with CKD have abnormal vascular remodeling that is a risk factor for cardiovascular disease. MicroRNAs (miRNAs) control mRNA expression intracellularly and are secreted into the circulation; three miRNAs (miR-125b, miR-145 and miR-155) are known to alter vascular smooth muscle cell (VSMC) proliferation and differentiation. We measured these vascular miRNAs in blood from 90 patients with CKD and found decreased circulating levels with progressive loss of eGFR by multivariate analyses. Expression of these vascular miRNAs miR-125b, miR-145, and miR-155 was decreased in the thoracic aorta in CKD rats compared to normal rats, with concordant changes in target genes of RUNX2, angiotensin II type I receptor (AT1R), and myocardin. Furthermore, the expression of miR-155 was negatively correlated with the quantity of calcification in the aorta, a process known to be preceded by vascular de-differentiation in these animals. We then examined the mechanisms of miRNA regulation in primary VSMC and found decreased expression of miR-125b, 145, and 155 in VSMC from rats with CKD compared to normal littermates but no alteration in DROSHA or DICER, indicating that the low levels of expression is not due to altered intracellular processing. Finally, overexpression of miR-155 in VSMC from CKD rats inhibited AT1R expression and decreased cellular proliferation supporting a direct effect of miR-155 on VSMC. In conclusion, we have found ex vivo and in vitro evidence for decreased expression of these vascular miRNA in CKD, suggesting that alterations in miRNAs may lead to the synthetic state of VSMC found in CKD. The decreased levels in the circulation may reflect decreased vascular release but more studies are needed to confirm this relationship.

## Introduction

MicroRNAs (miRNAs) are small (approximately 22 nucleotide), non-coding RNAs that function to regulate messenger RNAs (mRNAs) post-transcriptionally by degrading or repressing mRNA. miRNAs play a critical function during development through effects on cell proliferation, differentiation, and apoptosis [1], [2]. In the adult, abnormalities in miRNA have been identified in multiple disease states, including malignancies, inflammatory diseases, and cardiovascular diseases [1], [3]. The miRNA are further processed in the nucleus through activity of DROSHA, an RNAse III, into precursor miRNA that are transported into the cytoplasm. There they are cleaved by DICER to generate miRNA that are incorporated into the RNA-induced silencing complex (RISC) to form a RISC-miRNA complex that represses mRNA transcription or enhances mRNA degradation[3]. It is estimated that there are up to 1000 human miRNA, and that the majority of mRNA are potential targets of miRNA. Furthermore, each miRNA may affect multiple mRNA, and each mRNA may have multiple miRNA regulating its transcriptional activity [4], [5]. The miRNA are resistant to degradation, and in addition to their cellular effects, are released into the circulation and thus may also serve as circulating biomarkers[5], [6]. For example, in non-CKD patients with cardiovascular disease, miR-145 and miR-155 were found to be lower in patients with coronary artery disease than in those without [7].

Chronic kidney disease (CKD) is a known cardiovascular risk factor, and most patients with CKD die of cardiovascular disease before reaching the need for dialysis[8]. Once on dialysis, cardiovascular disease (CVD) accounts for 30% of hospitalizations and 50% of the mortality[9]. CKD is associated with a high prevalence of hypertension and diabetes, explaining some of this increased risk. However, non-traditional risk factors such as Chronic Kidney Disease-Mineral and Bone Disorder (CKD-MBD)[10]–[12] and inflammation[13], [14] have also been strongly associated with morbidity and mortality in CKD. Abnormal gene expression and cellular function due to derangements in miRNA expression may be one, as yet, unexplored mechanism for the pathogenesis of increased CVD risk in CKD. Therefore, in the present study we evaluated three “vascular” miRNAs that are known to be expressed in the artery and involved in vascular smooth muscle cell (VSMC) differentiation (miR-145 and 155)[2], [15], [16], inflammatory vascular disease (miR-155)[17]–[19], abnormalities in the angiotensin pathway (miR-155) [20]–[22] and arterial calcification (miR-125b)[23], [24] in patients with CKD. All of these vascular miRNAs have been shown in animal models to regulate VSMC de-differentiation, resulting in a more synthetic/proliferative phenotype that pre-disposes to altered remodeling response to stress. We assessed the circulating levels in CKD patients, the tissue expression in arteries ex vivo from CKD rats compared to normal animals, and the regulation of expression in cultured VSMC from CKD rats.

## Materials and Methods

### Human study

#### Ethics Statement

The study was approved by the IUPUI Institutional Review Board. All participants gave written informed consent with documentation to that effect in accordance with IUPUI standard operating procedures. (IRB#0707-04 and IRB#0011-48).

To determine if CKD affects the expression of circulating miR-125b, miR-145 and miR-155, we analyzed the expression in stored sera from 90 stage 3–4 CKD patients who had participated in a previous study and consented for future use of their blood samples. Inclusion criteria were stage 3–4 CKD (defined as estimated glomerular filtration rate [eGFR] calculated from 4-parameter MDRD equation ranged by 15 – 59 ml/min/1.73 m^2^), hemoglobin >10 g/dL, age >18 years, no evidence of active infection or inflammatory process, and not hospitalized within the previous 30 days. Subjects were determined to have atherosclerotic vascular disease (coronary artery disease, cerebrovascular accident, or peripheral vascular surgery/amputation) by self- report at the time of sample collection and/or retrospective chart review. The presence of left ventricular hypertrophy (LVH) was documented from echocardiographic reports performed within 2 years before or after the sample collection. The sera had been frozen at –80°C for an average of 25.3±6.8 months in aliquots with no previous freeze-thaws. To confirm that the storage had no effect on expression and to compare more diverse levels of kidney function, we also prospectively collected sera from 10 stage 3 and 4 CKD patients, 10 hemodialysis patients and 8 healthy volunteers and analyzed the samples within one month.

### Animal models and tissue harvest

To confirm that these vascular miRNA may be indicative of underlying tissue abnormalities, we utilized existing tissue samples from prior experiments with the Han:SPRD Cy/+_IU_ rat with polycystic kidney disease (ADPKD) [25]. This is an autosomal dominant condition, such that at birth, 1/4 of the animals are normal (+/+), 1/2 are heterozygotes (Cy/+), and 1/4 are affected homozygotes (Cy/Cy). Homozygous Cy/Cy rats develop massively enlarged kidneys and severe azotemia, and normally die by 4 weeks of age. The male Cy/+ rat develops a persistent azotemia starting at about 10 weeks of age, which progresses to terminal uremia by about 40 weeks, with spontaneous and slow development of all three manifestations of CKD-MBD: biochemical abnormalities, extraskeletal calcification, and abnormal bone[25]. Weaned rats were housed in open top, shoebox cages, and had free access to tap water and standard chow until they were 24 weeks old when they were switched from a standard pellet rat chow to a powdered diet of 18% casein-based protein, 0.7% phosphorus, 0.7% calcium, 5% fat (Harlan Teklan TD.04539). The animals (Cy/+ rat and normal littermate +/+, hereafter called CKD and Normal rat) were sacrificed at 35 weeks with pentobarbital (50 mg/kg intraperitoneally) and thoracic aortas were weighed and collected and stored at –80°C for RNA isolation. To quantify aorta calcification, proximal segments of thoracic aortas from normal or CKD animals were snap frozen. The aorta section was then weighed and incubated in 0.6N HCl for 48 hrs. The samples (n = 8 per group) were then homogenized, centrifuged, and the supernatant analyzed for calcium using the *o*-cresolphthalein complex one method (Calcium kit; Pointe Scientific) as previously published[26].

All procedures were reviewed and approved by the Indiana University School of Medicine Institutional Animal Care and Use Committee.

### RNA isolation and quantification

Total RNA from normal or CKD rat thoracic aorta was isolated using miRNeasy Mini Kit (Qiagen) according to the manufacturer’s instruction. Similarly, total RNA from human serum (400 ul) or cell lysate was isolated using the same kit. Total RNA was eluted from the column in RNase-free water and stored at –80°C. Quantification of miRNA was performed at the Center for Genetics in Indiana University School of Medicine using Agilent 2100 Bioanalyzer Small RNA kit.

### Cell culture

To determine potential mechanisms for our tissue findings, rat vascular smooth muscle cells (RVSMC) were isolated from descending thoracic aorta of the Han:SPRD Cy/+_IU_ (CKD) or normal rats by the explant method as previously described [27]. Cells were used at passage 3–5 and grown in Dulbecco’s Modified Eagle’s Medium (DMEM; Sigma, St. Louis, Mo., USA) containing 10% fetal bovine calf serum (FBS) until confluent at which time they were cultured in DMEM with 15% FBS, 10 mM of sodium pyruvate, 100 mM of insulin, and 50 ug/ml of ascorbic acid for various times. Cell lysates were homogenized and collected in QIAzol Lysis Reagent (Qiagen) according to manufacturer’s protocol and kept at -80°C until RNA isolation.

### Real time PCR

miRNA expression was determined by real time PCR using TaqMan miRNA assays (Applied Biosystems, Foster City, CA). Forty ng of total RNA were used for reverse transcription to synthesize complementary DNA using TaqMan miRNA-specific primers and the Taq reverse transcription kit (Applied Biosystems, Foster City, CA). Target-specific PCR primers (miR-125b, miR-145, miR-155 and miR-210) were obtained from Applied Biosystems. Real-time PCR amplifications were performed using TaqMan miRNA Assays (TaqMan MGP probes, FAM dye-labeled) using Applied Biosystems 7500 Real-Time PCR systems (Applied Biosystems). The cycle number at which the amplification plot crosses the threshold was calculated (C_T_), and the ΔΔC_T_ method are used to analyze the relative changes in gene expression and normalized by U6, a non-human ubiquitous miRNA. We further tested for intra-patient coefficient of variance (CV) by running PCR from serum collected from a single patient during 8 different RT-PCR runs, and demonstrated the inter assay CV for the sera was 3.8%, 2.0% and 4.7% for miR-145, miR-155 and U6 SnRNA, respectively. Thus, there was no evidence that uremic sera altered the CV% and thus we utilized the standard control U6 as an internal control. The expression of angiotensin II type I receptor (AT1R), myocardin, RUNX-2, Drosha and Dicer was also analyzed by real time PCR using Taqman gene expression assay system (Applied Biosystems) and normalized by beta-actin [27].

### Transfection

To confirm the role of miR-155 on its target gene AT1R in VSMC, a miR-155 mimic and miR negative control (Applied Biosystems) were used to promote the function of miR-155. Briefly, VSMC were seeded in 24-well culture plates until they were 60-70% confluent. Transfection of 30 nM of miR-155 mimic or negative control was performed using Lipofectamine RNAiMAX reagent (Applied Biosystems) according to the manufacturer’s instructions. Non-transfected VSMC was used as control. The over expression of miR-155 in VSMC was confirmed by real time PCR of total RNA isolated from miR-155 mimic or negative control transfected VSMC. The miR-155 target gene AT1R expression in VSMC was also determined by real time PCR.

### Cellular proliferation assay

VSMC were transfected with miR-155 mimic or negative control and cellular proliferation determined at 48, 72 and 96 hrs after transfection by Cell Titer 96 Proliferation Assay Kit (Promega Corporation) according to manufactures’ instruction. In addition, non-transfected VSMC were also used as control.

### Statistical Analysis

All patient variables were described as mean ± SD. For the human studies, review of circulating miRNAs, the miR-145 and miR-155 indicated the results were not normally distributed, and therefore all of the miRNAs were converted to natural logarithmic values. T-tests, tests of ANOVA or correlations were calculated to compare miRNA levels between categorical and continuous predictors, respectively. Predictors related to miRNA levels at significance levels of less than 0.1 were included in a multivariate linear regression models. Gender, race and MDRD eGFR were forced into the model. Two models were run, the first with all 85 subjects but no LVH, and the second model with only the 59 subjects that had an echocardiogram. For the animal ex vivo data and in vitro cell culture, comparisons between CKD and normal animals was done by t-test, and correlations by Pearson Product Coefficient with a p<0.05 considered significant. (StatView, SAS Institute, Cary, NC and SigmaPlot, Systat Software Inc, San Jose, California).

## Results

### Circulating levels of miR-125b, miR-145 and miR-155 are decreased in CKD

To determine if patients with CKD have low circulating levels of miRNA, we analyzed 90 stored blood samples of stage 3 and 4 CKD patients. The characteristics of the patients are shown in Table 1. By univariate analysis (p<0.1), the level of miR-125b was correlated with eGFR by MDRD, creatinine, calcium, 1,25(OH)_2_D, 25(OH)D, and diabetes; miR-145 with eGFR, diabetes, 25(OH)D, 1,25(OH)_2_D; and miR-155 with eGFR and diabetes. There was no relationship of any of these miRNA with CRP, vascular disease or LVH. However, vascular disease was associated with increased age (r = 0.21; p = 0.049) and diabetes and CRP were negatively correlated with presence of LVH (r = 0.22; p = 0.046). After multivariate adjustment, only eGFR by MDRD was associated with all three miRNA. For every one ml/min decrease in eGFR, there were a 0.05, 0.04, and 0.02 unit decreases in the expression of the miRNA for miR-125b, 145, and 155, respectively. In addition, after adjustment, diabetes continued to be associated with miR-145 (β = 1.69, p = 0.02, 95% CI 0.34, 3.04) but not miR-155. Calcium and vitamin D levels were no longer significant for miR-125b, miR-145 or miR-155. A model run only with those subjects who had undergone an echocardiogram (n = 63) failed to find any relationship with any of the miRNAs and LVH.

**Table 1:** Characteristics of stage 3–4 CKD patients (n = 90).

Characteristics	Mean ± SD
Age [years]	63.0±14.4
Gender (Male [n(%)])	51 (56.6)
Race (Caucasians : African Americans : Others)	51 : 38 : 1
Diabetes mellitus [n(%)]	48 (53.3)
Hypertension [n(%)]	89 (98%)
Vascular disease [n(%)]	33 (36.7)
LVH (no: yes: not done)[n)]	38:25:27
Serum creatinine (mg/dL)	2.0±0.7
4-parameter MDRD (ml/min/1.73 m^2^)	35.7±11.0
Albumin (g/dl)	3.6±0.4
Phosphorus (mg/dl)	3.5±0.7
Calcium (mg/dl)	9.3±0.4
Intact PTH (pg/ml)	118±58
25 hydroxy-vitamin D (ng/ml)	22±13
1,25 dihydroxy-vitamin D (pg/ml)	34±14
C-reactive protein (mcg/L)	7.4±8.1

**Figure 1:**
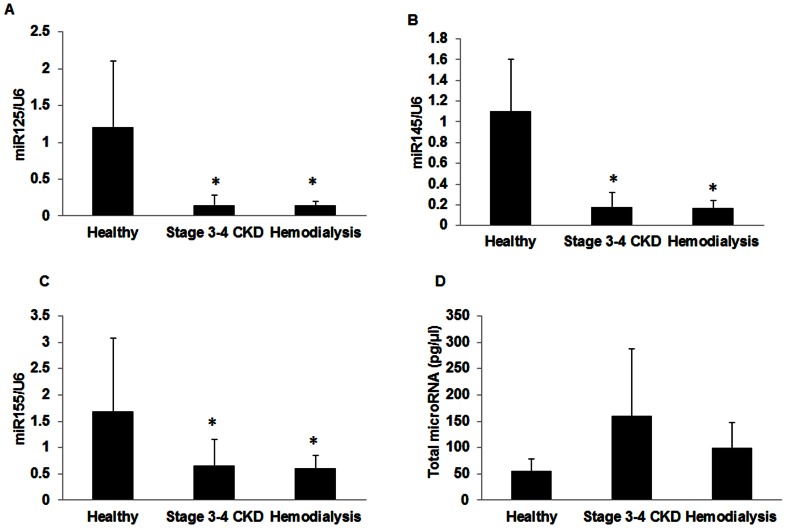
Circulating miRNA levels in controls, CKD patients and hemodialysis patients in freshly isolated samples. Sera were collected from stage 3–4 CKD patients (n = 10), hemodialysis patients (n = 10) and healthy volunteers (n = 8) and total RNA isolated and real time PCR performed to determine the expression of circulating levels of miR-125b (A), miR-145 (B) and miR-155 (C) normalized by U6. Total serum miRNA concentration in the three groups was measured using Agilent Bioanalyzer (D) to demonstrate that the proportion of miRNA to total RNA analyzed is not the etiology of decreased expression of these specific miRNA. Each sample was assayed in triplicate. Data were expressed as mean ± SEM. * p<0.01 compared to healthy volunteers; ** p<0.05 compared to healthy volunteers.

Given the above results we further examined the effect of the level of renal function on circulating levels of miR-125b, 145, and 155 by comparing freshly collected serum by real time-PCR from patients with stage 3–4 CKD, hemodialysis patients and healthy volunteers. The samples were collected, immediately frozen in aliquots and analyzed within a one month period, ruling out degradation with freezer storage. The clinical characteristics are shown in Table 2 and the results in Figure 1. The circulating levels of miR-125b (Figure 1A), miR-145 (Figure 1B) and miR-155 (Figure 1C) were all significantly decreased in CKD and hemodialysis patients compared to those in healthy volunteers. Because the samples were isolated and PCR run as total RNA, we further examined the actual concentration of miRNA using an Agilent Bioanalyzer. For equal amounts of total RNA, the proportion of miRNA in the samples was not statistically different, with a trend towards lower proportion in the healthy controls (Figure 1D). Therefore our low specific miRNA levels are not due to a decreased proportion of miRNA to total RNA in the circulation of patients with CKD, nor due to loss from degradation in the freezer.

**Table 2:** Patient characteristics in a cohort of freshly isolated miRNA samples.

Characteristics	Healthy (N = 8)	Stage 3–4 CKD (N = 10)	Hemodialysis (N = 10)
Age	43.4±11.5	61.1±14.1^a^	54.8±14.0^a^
Gender (%Male)	25	40	60
Race-Caucasians [N(%)]	5 (62.5)	10 (100)	1 (10)
Race-African Americans [N(%)]	0 (0)	0 (0)	9 (90)a,b
Race-Others [N(%)]	3 (37.5)	0 (0)	0 (0)
DM (%)	0	20	50
HbA1C (%)	–	7.85±2.05	5.88±0.55

a p<0.05 vs. healthy subjects; ^b^ p<0.01 vs. healthy subjects.

### The decreased expression of miRNAs in aorta is associated with changes in target gene expression and vascular pathology in CKD animals

**Figure 2:**
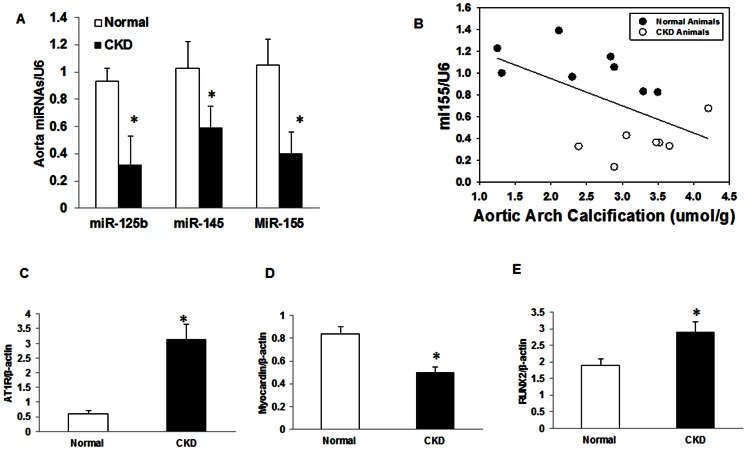
Decreased expression of miRNAs and downstream genes in thoracic aorta from CKD animals. Thoracic aorta were collected at 35 weeks of age from CKD (n = 8) and normal rats (n = 8) and total RNA was isolated and miRNA expression determined by real time PCR and normalized by U6. The results demonstrated that there is significantly reduction in expression in miR-125b, miR-145, and in thoracic aorta from CKD compared to that from normal rats (Figure 2A). Aortic calcification was determined biochemically and results demonstrated that aorta calcification is increased in CKD rats and the greater the calcification, the lower the miR-155 expression level (r = 0.54, p = 0.04; Figure 2B). Real time PCR was also performed to determine the expression of several target genes and normalized by β-actin. The results demonstrated that the expression of AT1R (Figure 2C) and RUNX2 (Figure 2E) is increased whereas myocardin expression is decreased (Figure 2D) in aorta from CKD rats compared to normal rats, corresponding to known physiologic roles of these vascular miRNA. Data were expressed as mean ± SEM. * p<0.05, CKD vs. normal.

In order to determine if the miRNA identified in the sera of patients with CKD had biologic plausibility as potential biomarkers, we utilized previously collected thoracic aorta tissue harvested at 35 weeks of age from CKD and normal animals. There was no difference in total miRNA concentration between normal and CKD rats (216±61 vs. 221±72 ng/ul; Normal vs. CKD). By real time PCR, the expression of miR-125b, miR-145 and miR-155 were lower in thoracic aorta from CKD compared to that from normal rats (all p<0.01; Figure 2A). As we have previously reported [25], there was increased calcification in the aorta arch of the CKD animals compared to the Normal animals (3.3±0.6 vs. 2.4±0.8 umol/g tissue, p = 0.04). To determine the relationship between calcification and down regulation of the miRNA, we compared the correlation between the magnitude of calcification and concentration of the miRNA. There were negative correlations of miR-155 with the presence of calcification miR-155 (r = –0.537, p = 0.04; Figure 2B) and miR-125b and calcification (r = –0.478, p = 0.07), but no relationship with miR-145. We then examined downstream gene products that are known to be regulated by these miRNA. For example, miR-155 directly targets angiotensin II type I receptor (AT1R) and inhibits AT1R expression. On the other hand, miR-145 controls VSMC phenotype by increasing target gene myocardin expression via inhibition of an intermediary [20], [28]. The results found that the expression of angiotensin II type I receptor (AT1R) was increased (Figure 2C) and myocardin was decreased (Figure 2D) in CKD animals compared to Normal animals. Furthermore, there was a strong negative correlation between the miR-155 and expression of AT1R (r = –.078, p<0.001) and myocardin (r = –0.608, p = 0.02). However, there was no correlation between AT1R and RUNX2, or between miR-145 and myocardin. The expression of RUNX2 was also increased in CKD animals (Figure 2E), with a non-statistical correlation of RUNX2 with miR-125b (r = –0.47, p = 0.07) and miR-155 (r = –0.45, p = 0.08). In summary, compared to Normal animals, the CKD animals had increased calcification in the thoracic aortas, decreased expression of these vascular miRNAs, and a corresponding increased expression of RUNX2, AT1R, and decreased expression of myocardin.

### The expression of vascular miRNAs is decreased in VSMC from CKD compared to normal animals

**Figure 3:**
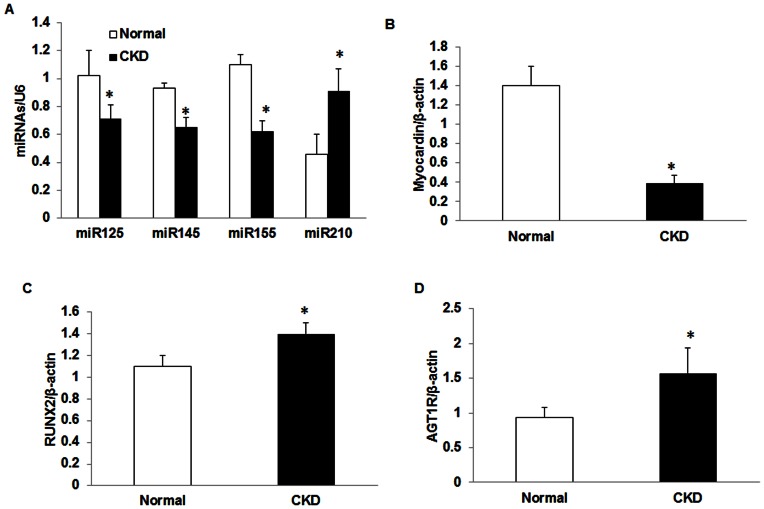
The Expression of vascular microRNAs and target genes in VSMCs from normal and CKD rats. Rat VSMC isolated from normal or CKD rats were cultured in growth media for 4 days and total RNA isolated. Real time PCR was performed to determine the expression of miR-125b, miR-145, miR-155 and miR-210 and normalized by U6 (A). The expression of myocardin (B), RUNX2 (C) and AT1R (D) was also determined by real time PCR and normalized by β-actin. Each sample (n = 9 with cells isolated from 3 different normal or CKD animals) was assayed in triplicate. Data were expressed as mean ± SEM. * p<0.05, CKD vs. normal.

To determine the effect of CKD on miRNA expression, we isolated VSMC from the aorta of CKD (Cy/+) rats and their normal littermates and determined the expression of miR-125b, miR-145, and miR-155 by real time PCR. Cultured VSMC from CKD rats had significantly lower expression of miR-125b, miR-145 and miR-155 compared to that from normal rats (Figure 3A). We also analyzed miR210, a miRNA known to be upregulated in acute kidney injury[29], [30], demonstrating increased expression in the VSMC from our CKD animals (Figure 3A), confirming our findings were not due to uniform miRNA suppression. Furthermore, the total miRNA concentration in these samples from normal and CKD rats was not different (normal 253±126 pg/µl; CKD 312±91 pg/µl). Cellular miRNA are processed from pro-miRNA by DROSHA and DICER and thus we determined the expression of these enzymes in VSMC from CKD compared to normal rats. The results demonstrated no difference in the expression of DROSHA/β-actin (normal 1.27±0.20; CKD 1.28±0.25) and DICER/β-actin (normal  = 1.05±0.01; CKD  = 1.0±0.06) in VMSC between normal and CKD rats. Similar to that observed in ex vivo aorta samples, we found a decrease in myocardin (Figure 3B) and an increase in expression of RUNX-2 (Figure 3C) and AT1R (Figure 3D) in CKD VSMC compared to normal VSMC.

### Effect of overexpression of miR-155 on AT1R expression and cellular proliferation in VSMC

**Figure 4:**
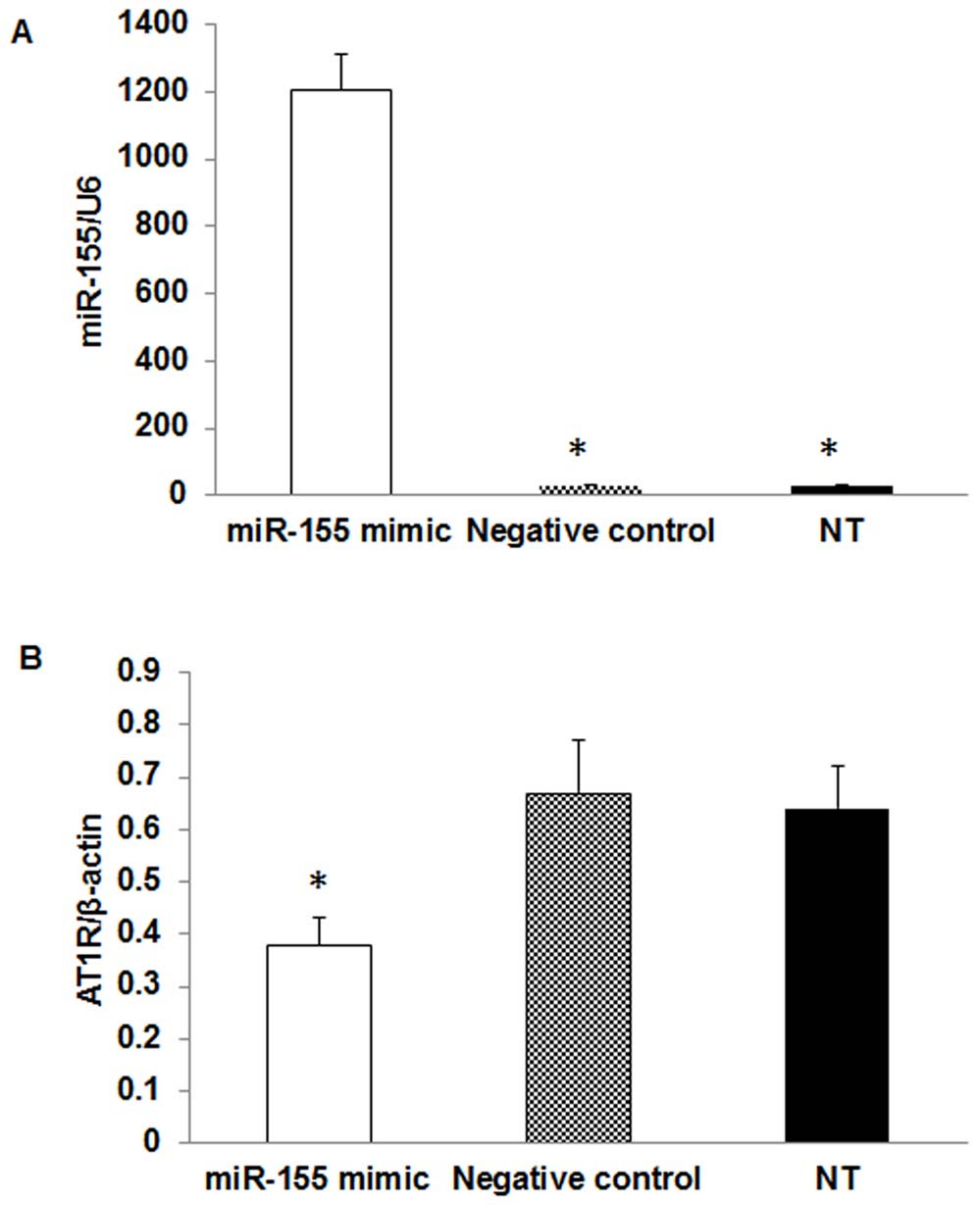
Overexpression of miR-155 and AT1R expression in VSMC from CKD rats. Rat VSMC isolated from CKD rats were transfected with 30 nM of miR-155 mimic or miR negative control for 48 hrs. Non-transfected VSMC (NT) was also used as control. The overexpression of miR-155 in VSMC was confirmed by real time PCR (A). The miR-155 target gene AT1R expression in VSMC was also determined by real time PCR, demonstrating a significant inhibition of AT1R expression compared to that with negative control or non-transfected VSMC (B). Data were expressed as mean ± SEM (n = 3 separate experiments). *p<0.05, miR-155 mimic vs. negative control or NT.

**Figure 5:**
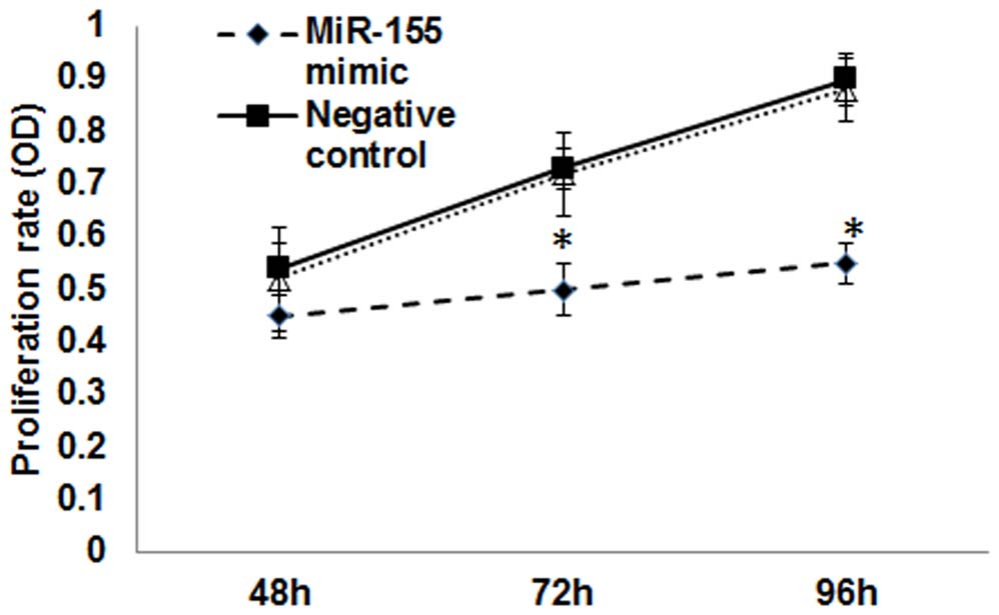
Upregulation of miR-155 decreases cellular proliferation in VSMC from CKD rats. Rat VSMC isolated from CKD rats were transfected with 30 nM of miR-155 mimic or miR negative control. Non-transfected VSMC (NT) was also used as control. The cellular proliferation was determined 48, 72 and 96 hrs after transfection using Cell Titer 96 Proliferation Assay Kit. The results demonstrated that the transfection of miR-155 mimic significantly inhibited cellular proliferation at 72 and 96 hrs in VSMC from CKD rats compared to that with negative control or non-transfected VSMC. Data were expressed as mean ± SEM (n = 3 separate experiments). *p<0.05, miR-155 mimic vs. negative control or NT.

To confirm a direct relationship between the levels of miR-155 on target gene AT1R expression, VSMC from CKD rats were transfected with miR-155 mimic or miR negative control for 48 hrs. The results demonstrated that compared to negative control or non- transfected cells, VSMC transfected with miR-155 mimic had more than 1000 fold increase in miR-155 expression (Figure 4A). Furthermore, the AT1R expression in VSMC transfected with miR-155 was significantly decreased compared to that negative control or non-transfected VSMC (Figure 4B). The change in phenotype do a differentiated VSMC is usually accompanied by increased proliferation [31], we therefore examined the effect of miR-155 on proliferation in VSMC from CKD rats. As shown in Figure 5, cellular proliferation were significantly inhibited at 72 and 96 hrs in VSMC transfected with miR-155 mimic compared to that in miR negative control or non-transfected VSMC. These results suggest that miR-155 directly controls the AT1R expression and regulate cellular proliferation in VSMC.

## Discussion

**Figure 6:**
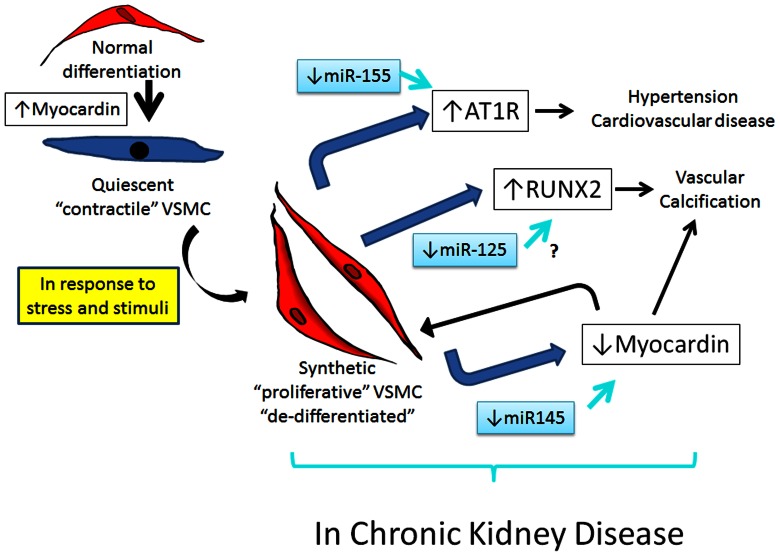
Hypothesis of the impact of vascular miRNAs on the pathogenesis of cardiovascular disease of CKD. This figure is our hypothesis of how the miRNAs may affect cardiovascular disease in CKD. During development, normal differentiation of VSMC is controlled in part by the ‘master’ regulator, myocardin. In adulthood, the majority of VSMC are in a quiescent, synthetic state. During acute insults, these synthetic VSMC change to a more proliferative state, returning to a quiescent state after the insult. However, in the setting of kidney disease, VSMC appear to stay in a more proliferative state with corresponding increased expression of AT1R and RUNX2, and decreased myocardin expression. The decreased expression forces the VSMC to remain in a continual proliferative or de-differentiated state. MiRNAs are known to be important in regulating such differentiation during development, but the low levels of miR-155, 145, and 125b observed in CKD arteries and VSMC and in the circulation of patients with CKD in the present study may lead to further propagation of de-differentiated VSMC, potentiating the development of hypertension, cardiovascular disease and vascular calcification.

Patients with CKD are more likely to die of a cardiovascular event than progress to dialysis[8] and once on dialysis cardiovascular disease is the leading cause of death. We chose to examine miR-125b, 145 and 155 due to their known role in the regulation of the VSMC phenotype and the finding that circulating levels are decreased in patients with coronary artery disease[7]. In the present study we demonstrate that these vascular miRNAs are decreased in the serum of patients with CKD and hemodialysis patients compared to controls and therefore are potential biomarkers. In a cohort of 90 patients, these decreased levels were correlated with various abnormalities of CKD, but by multivariate analysis only loss of kidney function was associated with decreased circulating levels of all three miRNAs. To determine the biologic plausibility that these markers may reflect underlying pathology, we utilized animals and demonstrated that, compared to normal animals, miR-125b, 145 and 155 are decreased in the thoracic aorta ex vivo from CKD animals and cultured VSMC from CKD animals. This model of CKD, the Cy/+ rat is known to have progressive CKD, increased left ventricular hypertrophy, arterial calcification and hypertension [25], [32] and thus are phenotypically similar to patients with advanced CKD. We further examined the mechanism by which these miRNA of vascular origin are decreased by comparing expression and regulation in VSMC from CKD rats compared to normal littermates. The results demonstrated normal processing enzyme expression of DROSHA and DICER, but decreased expression of all three vascular miRNA in VSMC from CKD animals compared to normal animals. Unfortunately we were unable to measure miRNA in the circulation of rats in which tissue samples existed due to the methods used to collect the samples. These data support that the low levels of these miRNA will lead to ‘unregulated’ mRNA and phenotypic vascular changes in the setting of CKD in our animal model. Such phenotypic switch to a more proliferative/synthetic state is important in the aberrant remodeling of CKD and in vascular calcification (Figure 6) [33], [34]. Indeed, we found that downstream mRNA products directly regulated by these vascular miRNAs were also affected. AT1R is normally repressed by miR-155, and thus our findings of increased AT1R expression in the arteries and VSMC of CKD animals may represent a defect in ‘regulation’ by the low levels of this miRNA. Using transfection techniques, we confirmed a direct regulatory effect of miR-155 on AT1R expression in VSMC from CKD rats. Thus, the known upregulation of AT1R in arteries of patients with CKD [35] may be a direct result of low levels of the controlling miRNA-155. Furthermore, we demonstrated that overexpression of miR-155 inhibited cell proliferation, confirming that the low expression of miR-155 may be a causative factor in the proliferative VSMC state observed in CKD. Although many significant correlations were observed, the most consistent observation was with miR-155, whose expression was negatively correlated with vascular calcification. Taken together, the data strongly supports a role of miR-155 in the aberrant vascular remodeling in CKD. Time dependent changes should be evaluated in the future.

The phenotype of proliferative VSMC has been linked to hypertension, aberrant response to remodeling after angioplasty, atherosclerosis and vascular calcification, all common problems in patients with CKD (Figure 5). Myocardin is a “master regulator” of VSMC phenotype that is upregulated during normal development/differentiation, but becomes down regulated when VSMC turn from a quiescent/contractile state to a proliferative/synthetic state [36]. Myocardin is normally stimulated by miR-145 [28] and thus, the low expression of myocardin in our study confirmed the functional importance of the decreased level of miR-145. Furthermore, another marker of vascular de-differentiation to an osteoblast phenotype, RUNX-2, was increased. Thus, in addition to the effects of miR-155, these results suggest that CKD is associated with decreased VSMC expression and decreased circulating levels of several miRNA that control VSMC differentiation and function. The sustained suppression of myocardin would further lead to a protracted proliferative state of VSMC. Whether these abnormalities are the cause of vascular disease in CKD, or due to the presence of vascular disease will need to be determined from future in vivo studies.

Several other studies support our findings that altered regulation of these vascular miRNAs are important in the aberrant vascular disease of CKD. In spontaneously hypertensive rats, the tissue expression of miR-155 in the aorta is inversely related to blood pressure [22]. Aortic adventitial fibroblasts (often called myofibroblasts) with decreased miR-155 had decreased Angiotensin II receptor expression [21], a finding that parallels our observations. Furthermore, overexpression of miR-155 led to differentiation to a more contractile myofibroblast[21]. MiR-155 inhibits human angiotensin type 1 receptor (AT1R) expression and the activation of AT1R receptor mediated signaling cascades in fibroblasts[20]. In addition, one polymorphism in AT1R is likely significant due to alteration in the miR-155 binding site[37]. Thus, low levels of circulating miR-155 may lead to activation of AT1R in patients with CKD, perhaps playing a role in cardiovascular disease and renal fibrosis [35]. A recent study linked low circulating miR-155 levels with increased risk of sudden cardiac death [38], a leading cause of death in ESRD patients. miR-155 is also important in inflammation, and is expressed in both activated B and T cells[18], perhaps further adding to the pathogenesis of atherosclerosis. Importantly, not all miRNA were downregulated in the CKD VSMC as miR-210, previously shown to be upregulated in acute kidney injury[30], was upregulated in CKD VSMC compared to normal littermates. Thus, the decreased miR-155 levels in the arteries of our CKD rats compared to normal littermates are consistent with the known end organ manifestations of hypertension, left ventricular hypertrophy and coronary artery and aorta calcification in these CKD animals[25], [32].

VSMC differentiation is tightly controlled during development, but VSMC maintains plasticity such that de-differentiation occurs in response to various stimuli in adult life. The regulation of this de-differentiation is thought to play a major role in pathogenesis of hypertension, arteriosclerosis and arterial calcification in CKD [33], [34]. Elia et al have shown that the expression of miR-145 is decreased in mice subjected to aortic constriction and in the ApoE mouse model of atherosclerosis. Furthermore, they demonstrated that VSMC from miR-145 null mice are de-differentiated [38]. Importantly, miR-143/145 complex regulates the major phenotypic regulator of VSMC myocardin such that decreased expression converts VSMC to a more proliferative, de-differentiated cell [2], [16]. Our in vitro work confirms that in the VSMC from CKD animals there was downregulation of both miR-145 and myocardin, indicating a more proliferative VSMC [2], [16]. Such down regulation of myocardin has also been observed in human VSMC transfected with siRNA to klotho[39], and with transfection of the receptor for advanced glycation end-products (RAGE)[40]. Furthermore, myocardin suppression is downregulated in vascular calcification [39], [41], although this may not be as critical as the upregulation of Runx2 in the VSMC ‘reprogramming’ towards osteochondrogenesis[42]. Since miR-145 normally increases myocardin, low levels observed in our study in VSMC in animals with CKD in vitro and in vivo, and in the circulation of patients with CKD may imply that the normal ‘safeguard’ regulation of myocardin by miR-145 may be impaired. More work is needed to confirm this hypothesis.

VSMC and osteoblasts differentiate from a common mesenchymal precursor, and the de-differentiation of VSMC to osteoblast like cells is thought to be an initiating factor in the pathogenesis of arterial calcification [42], [43]. We have previously demonstrated that high phosphorus, and uremic serum without elevated phosphorus concentrations, induced upregulation of the osteoblast transcription factor RUNX-2 and mineralization[44]. Furthermore, RUNX2 is also upregulated in vivo in areas of both intimal and medial calcification of arteries from ESRD patients[45]. The potential importance of miR-125b in the regulation of this RUNX2 mediated osteochondrogenic differentiation is supported by a study demonstrating that miR-125b inhibits the BMP-4 induced differentiation/proliferation of mesenchymal stem cells towards an osteoblast phenotype. When the activity of miR-125b was blocked, there was increased alkaline phosphatase activity and mineralization, suggesting continued osteoblast differentiation of these mesenchymal stem cells [23]. When VSMC[24] and adipocytes[40] are induced towards an osteogenic differentiation pathway through high phosphorus containing media, there is a decline in miR-125b expression. Finally, Goettsch et al found that miR-125b was decreased during calcification of human VSMC [24]. Thus, our findings of down regulation of miR-125b in both the circulation of patients with CKD and in arteries and VSMC from CKD rats, and an upregulation of RUNX2 in arteries/VSMC, suggests that miR-125b may play a role in preventing this de-differentiation of VSMC to osteoblast like cells. However, we did not find a significant correlation between miR-125b and RUNX2 expression. At this time there is no evidence that RUNX-2 is directly regulated by miR-125b and thus its effects are likely further upstream and/or earlier in the differentiation pathway[46]. Indeed, miR-125b has over 7000 possible gene targets (www.microRNA.org) and is involved in the regulation of NF-κB (nuclear factor kappa beta)[47] and thus controls differentiation in cells of multiple origins. Thus, the low levels of miR-125b observed in both the aorta tissue of CKD rats and the sera of CKD patients may have widespread effects on cell differentiation in multiple organ systems in CKD.

Despite the potential adverse consequences of low levels of these vascular miRNAs observed in our study using the rat model, we were unable to directly link these observations to circulating levels in the rat as correlating blood samples were not collected in a manner to allow miRNA analyses. Furthermore, we did not find an association of circulating levels with the presence of hypertension, vascular disease (defined broadly as coronary artery disease, peripheral vascular disease and/or stroke), nor left ventricular hypertrophy in our human study. This is in contrast to the work of Fichtlscherer et al who found that miR-145 and 155 were decreased in non-CKD patients with coronary artery disease with or without diabetes[7]. The differences may have been due to the very high prevalence of cardiovascular disease, some of which was likely undiagnosed, in our cohort. Alternatively, the miRNA levels in CKD may have been so low due to kidney disease, that this over shadowed any relationship with cardiovascular disease. It is also possible that measuring single miRNA may not provide adequate prediction. Recent studies clearly demonstrate that multiple miRNAs work in concert to regulate key physiologic function[48]. Thus, microarray methods may be more helpful to demonstrate an overall picture of alterations in miRNA in CKD patients. In contrast to the uniform decrease in specific miRNAs we found in our study, it is important to note that another group of investigators found that miR-499 was increased 80 fold in dialysis patients compared to controls; interestingly despite this marked elevation it appeared that standard hemodialysis substantially reduced levels (implying a profound increase in production)[49]. Clearly, additional studies linking blood levels to tissue levels in rats and human blood levels to outcomes prospectively are needed.

Our results are consistent with the study of Neal et al who demonstrated that miRNA levels similarly correlated inversely with decreasing GFR, and also found decreasing miR-155 with decreasing eGFR[50]. They hypothesized that this was due to degradation by circulating RNAse and performed an ex vivo study on exosomes from colonic cancer epithelium, demonstrating increased degradation when incubated with plasma from CKD patients compared to control plasma[50]. Thus, they did not evaluate if such degradation occurs in vivo. It is important to point out that the real time PCR is analyzed on equivalent amounts of total RNA isolated from blood. For this reason, we individually measured the actual quantity of miRNA in a randomly selected group of these samples and found an increase in the proportion of miRNA to total RNA in the samples from patients with CKD (Figure 3D) which would suggest there is not degradation in the circulation. We further tested freshly collected specimens and found similar results in our analyses. Therefore we believe it is unlikely that degradation could explain the low levels of the vascular miRNAs observed in our patients. Our in vitro work further supports that there is decreased synthesis. More work needs to be done to characterize the production, degradation and clearance of miRNA in CKD prior to their use as biomarkers.

In summary, we demonstrated the existence of circulating microRNAs in CKD and in dialysis patients. The circulating levels of miR-125b, miR-145 and miR-155 are decreased with progressive eGFR; the ability to detect such miRNA may offer hope of a novel cardiovascular biomarker. To determine the biologic plausibility of these miRNA serving as a future biomarker, we utilized an animal model and found the expression is decreased in aorta and cultured VSMC from CKD rats compared to normal littermates. Furthermore the decrease in and the tissue artery levels in rats corresponded to altered levels of mRNAs they are known to regulate, either directly or indirectly, and, at least for miR-155, correlated with arterial calcification. Upregulation of miR155 demonstrated a decrease in AT1R expression and decrease in proliferation, confirming a direct causative role of the low level of miR155 on VSMC to a more synthetic, proliferative phenotype. The mechanism for the decreased expression of these vascular miRNAs in CKD is unclear as we found normal expression of the miRNA processing enzymes DROSHA and DICER and no evidence for degradation in the circulation in CKD. Whether downregulation of these miRNAs are the cause of, or a consequence of, the widespread vascular phenotype abnormalities in our patients with CKD remains to be determined. In conclusion, CKD is associated with decreased expression of vascular miRNA in rats. Preliminary data in humans also show low circulating levels. Future studies are required to determine if the assessment of circulating levels in humans reflect underlying pathology.
